# Palliative Care Within the Primary Health Care Setting in Australia: A Scoping Review

**DOI:** 10.3389/phrs.2022.1604856

**Published:** 2022-09-06

**Authors:** Deborah van Gaans, Katrina Erny-Albrecht, Jennifer Tieman

**Affiliations:** Research Centre for Palliative Care, Death and Dying, College of Nursing and Health Sciences, Flinders University, Adelaide, SA, Australia

**Keywords:** public health, workforce, palliative care, primary healthcare, general practitioners

## Abstract

**Objectives:** This scoping review identifies and details the scope of practice of health professionals who provide palliative care within the primary health setting in Australia.

**Methods:** A scoping review approach was conducted on the Cinahl (Ebsco), Scopus, Medline (Ovid) and PubMed databases to extract articles from 1 December 2015 to 1 December 2020. Broad text words and MeSH headings were used with relevance to palliative care, general practice, primary health, and community setting. Extracted journal articles were limited to those based on the Australian population or Australian health system.

**Results:** Eighty-four papers met the inclusion criteria and were included in the review. The review identified the following health professional roles within the Primary Health Care setting undertaking palliative care: General Practitioner, Nurse, Pharmacist, Paramedics, Carers, and Allied Health professionals.

**Conclusion:** This review offers a first understanding of the individual health professional roles and multidisciplinary team approach to actively providing palliative care within the Primary Health Care setting in Australia.

## Introduction

In Australia, as in many other countries around the world, there is an increasing demand for palliative care services as a result of an aging population and an increase in the prevalence of cancer and chronic disease [1]. This has led to palliative care being provided in almost all health care settings within the Australian health care system, including hospital intensive care units, inpatient services, outpatient services, general practices, ambulatory services, pharmacies, and residential aged care facilities [2]. As a result, in Australia palliative care has become highly institutionalized [3]. However, recent survey data reveals that 60%–70% of Australians would prefer to die at home [4], which echoes the emerging trend of older Australian’s preferring to age in place. Though for this shift in the preferred place of death to occur within Australia, end-of-life care will have to change to provide more support for dying at home [3].

In response to the growing demand for palliative care at home, on the first of July 2021, the Australian Government provided funding (Greater Choice for At Home Palliative Care Measure) to the Primary Health Networks (PHNs), which are independent organizations focused on rationalizing health services to ensure equitable and appropriate health care provision [5]. The goals of the Greater Choice for At Home Palliative Care measure are to:

• improve your access to the best palliative care at home;
• support palliative care services in primary health and community care;
• make sure you get the right care, at the right time and in the right place to reduce unnecessary hospital visits;
• generate and use data to improve services;
• use technology to provide flexible and responsive care, including after-hours care [6].

PHNs are well placed to help coordinate at-home palliative care as their primary roles are to commission health services, work closely with health professionals to build health service capacity, integrate services at a local level, and ensure that health services meet the requirements of their region’s population [5].

While the Australian Government has provided financial incentive to develop palliative care within the Primary Health Care setting, many primary health care providers may not consider they have many palliative care patients even though they may provide care for people with progressive, chronic and/or life limiting illness and older people approaching the last years of life. This can make maintaining a depth of knowledge and acquiring new knowledge on palliative care difficult. In particular knowledge on recent trends in pharmaceutical management, new pain management, and approaches to using these medications are especially challenging [7]. Health professionals working in the Primary Health Care setting have also reported that their undergraduate education does not adequately cover palliative care and that they may not possess the skills required to manage palliative care patients [7]. This highlights the need by health professionals within the Primary Health Care setting for quality information and resources on palliative care management. Of high importance is the need for evidence and information to develop their professional knowledge, to enable clinical and service delivery of palliative care [8].

There is a growing body of evidence on providing Palliative care within the Primary Health Care setting and a need by Health Professionals to be able to access this evidence easily and quickly to provide best clinical practice and more generally for professional development [9]. However, providing access to appropriate information in a format that is feasible for health professionals is challenging [7]. There are several reasons for this, including the lack of time of health professionals have, as well as the volume of published research which makes specific evidence hard to find and manage [9].

The objective of this scoping literature review is to provide an overview of health professionals providing palliative care within the Primary Health Care setting within Australia. The results from this scoping review will then be developed into content for a new section within CareSearch, which provides health professionals, as well as those affected by the need for palliative care, with online evidence and information [10]. In this way, health professionals in the Primary Health Care setting are provided with information that will enable them to provide high-quality palliative care [11].

## Methods

### Search Strategy

We used a scoping review approach to collect and describe peer-reviewed journal articles from the last 5 years [12, 13]. Due to the relatively new field of palliative care within the Primary Health Care setting within Australia, a scoping review methodology was used rather than a systematic review, as it was better suited to clarifying key concepts and identifying available evidence.

Peer-reviewed journal articles were retrieved through searching electronic databases. The database search strategy (SupplementaryAppendix S1) was developed and tested in PubMed with the help of a health Librarian. Broad text words and MeSH headings were used: palliative care, general practice, primary health, Australia, allied health personnel, and community setting. Extracted journal articles were limited to those based on the Australian population or Australian health system. A date limit of the last 5 years was applied due to time and resource constraints. Once the search was finalised and run in PubMed, it was then translated and run in Cinahl (Ebsco), Scopus, and Medline (Ovid) on 1 December 2020.

### Study Selection

The search results for each database were uploaded into Endnote X9.3 reference management software and deduplicated. Journal articles were then imported into the web-based software Covidence for screening and data extraction.

Two reviewers (DvG and KEA) independently assessed titles and abstracts against the priori inclusion criteria outlined in Table 1. Where eligibility was unclear based on the title and abstract screening, the full text article was retrieved and assessed.

**TABLE 1:** Inclusion criteria for title and abstract screening of the included papers (Australia, 2022).

Criterion	Description
Palliative care	“Palliative care is person-centred and family-centred care provided for a person with an active, progressive, advanced disease, who has little or no prospect of cure and who is expected to die, and for whom the primary goal is to optimise the quality of life.” [14]
Australia	Australian population or Australian health system
Primary Health	Primary Health care setting
Types of studies	Quantitative or qualitative studies, including peer-reviewed journal articles, editorials, opinion pieces and case studies

Criteria for inclusion were agreed upon by both reviewers (see Table 1.)

Two reviewers (DvG and KE-A) then defined exclusion criteria (Table 2) to exclude articles which were not relevant to the aim of the scoping review. Both reviewers then screened the full text against the exclusion criteria.

**TABLE 2:** Exclusion criteria for title and abstract screening of the included papers (Australia, 2022).

Criterion
Not general practice/primary health/community setting
Not palliative care
Not Australian population or Australian health system
Conference abstract only
Tools
Duplicate
Other

## Results

**FIGURE 1:**
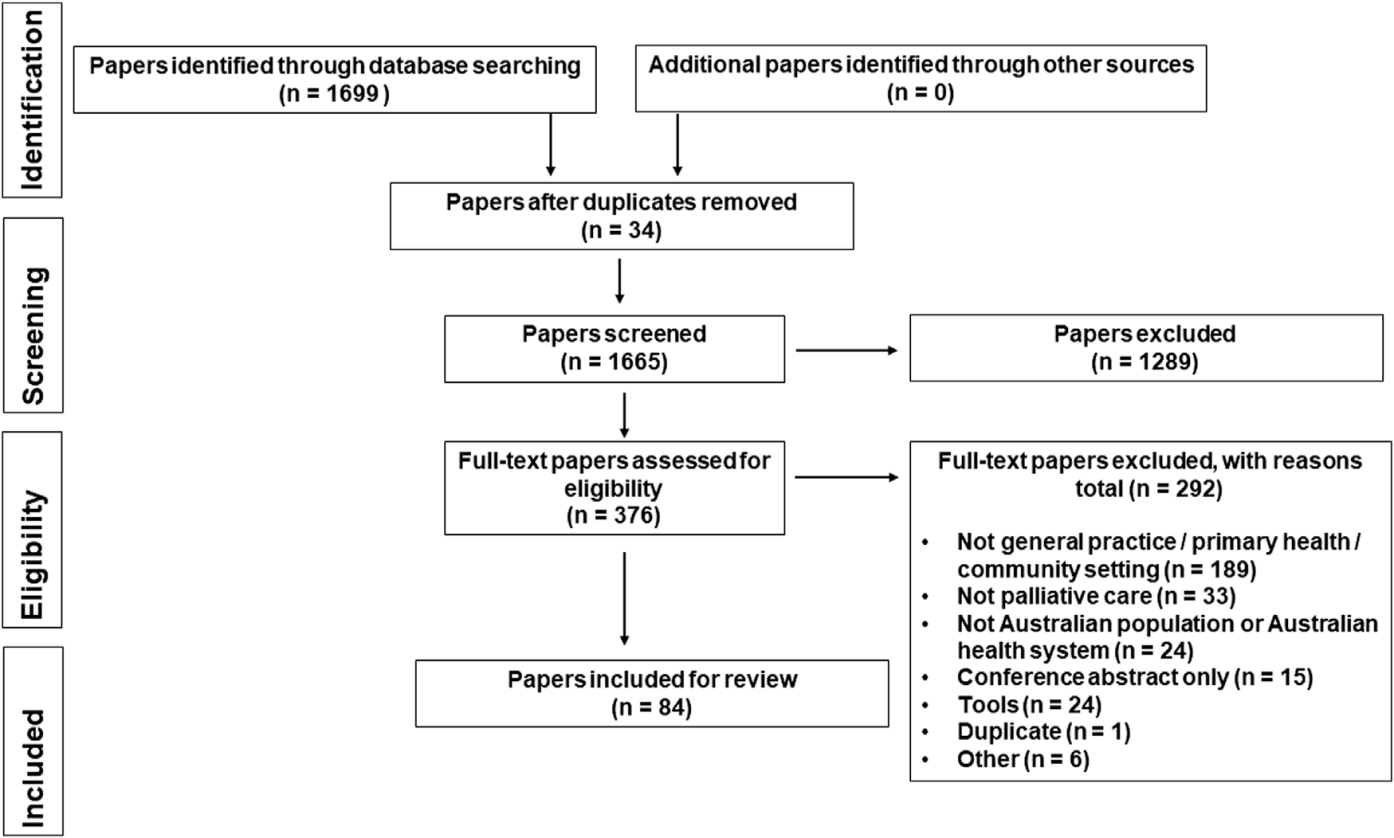
Search results in a PRISMA (Preferred Reporting Items for Systematic Reviews and Meta-Analyses) Flow Diagram (Australia, 2022).

The literature search strategy (Figure 1) highlights the systematic approach that was undertaken to select relevant publications from the initial 1,699 that were identified through the database searches. Covidence software [15] was then used to remove duplicate publications (*n* = 34) and review the publications title and abstracts against the inclusion criteria (Table 1) which resulted in the removal of 1289 publications. The full text of the remaining publications were then reviewed against the defined exclusion criteria and a further 292 publications were removed. The independent review process resulted in 84 publications being included in the scoping review (SupplementaryAppendix S2). Findings are presented for defined health professional roles.

### General Practitioners

As the population ages, GPs and General Practice Nurses are increasingly expected to provide end-of-life care [16, 17]. As a natural extension of primary care, palliative care often includes GPs providing continuity of care by setting up an individualized care pathway from diagnosis to palliation, coordinating care across that pathway, and collaborating with other health care providers [18–22]. Hence, most General Practitioners are already practicing end-of-life care without recognising it [23]. Since GPs provide care throughout a patient’s lifetime they are well positioned to identify those with life-limiting illnesses and to address the needs of patients and caregivers [20, 24, 25].

The palliative care process is very complex and involves interactions between GPs and patients and their carers as well as other service providers both in the community and in acute care settings [20]. GPs are essential players in an integrated model of palliative care as they are often intimately familiar with patients and caregivers, expert in generalist care, and knowledgeable about health and social services in their local region [18, 26]. Team members vary depending on patients’ needs and local availability of health professionals, but in most cases, home-based palliative care teams are led and coordinated by the GP [27]. Health professional members include nurses, social workers, occupational therapists, other allied health professionals, and the local pharmacist, however if the patient is more complex, specialist palliative care services may be enlisted by the GP to complement the skills of the local team [27]. Therefore community based palliative care is often seen as a collaborative, integrative model incorporating generalist and specialist palliative care providers that is coordinated by a GP [28], often through interprofessional case conferences [16].

The GP role is diverse and includes symptom management, pain management and non-malignant disease management [16]. The GP is proactive in clinical care planning, anticipating and providing care as the person’s clinical condition deteriorates and as the goals of care shift from treating illness to comfort and death management [29]. Therefore, the GPs role also includes making referrals to specialist palliative care services [25, 30]. GPs are also often involved in end-of-life conversations, starting with establishing a strong doctor-patient relationship, gauging patients’ readiness to engage, and managing time availability [31]. Conversations usually start when a patient initiates them, when they are included in routine care, when they discuss prognosis or less directly inquire about the patients feelings about death [31]. GPs commit time and resource to assist their patients to create an end-of-life care plan [32], however, at times the end-of-life care plan is not explicitly articulated and discussed, so an informal care plan is developed gradually without discussing these plans with the patient [33].

With limited specialist palliative care resources available in the community, it is often GPs who provide and co-ordinate end-of-life care in collaboration with community-based support services [18]. This has resulted in the GP role differing slightly in urban versus rural settings due to resource limitations and lack of training [34]. Although inappropriate payment models discourage the involvement of GPs in certain end-of-life care aspects, such as case conferences and home visits, rural communities often indicate closer relationships between GPs and patients and better care integration and collaboration [34]. This points out the importance of providing GPs and rural/remote communities with support, education, incentives, better administrative tools, and options for advanced care planning [35].

### Paramedics

Paramedics play an important role in community-based palliative care [36], their role is to treat patients who are experiencing health problems related to palliative care, such as pain and respiratory distress [37]. With Extended Care Paramedics responding to palliative care emergencies, and preventing unwanted and unnecessary hospitalisations [38]. In response to the increased use of Ambulance services in Australia to palliative care patients they are developing specific palliative care guidelines [37].

### Carers

Patients’ caregivers usually know them best and are most motivated to help [27]. In the Primary Health Care setting, a palliative patient’s caregiver can provide assistance with activities of daily living, personal and domestic care, and shopping and household responsibilities [39]. Assistance may also include basic nursing care, as determined by the patient’s GP and the specialist palliative care team [39]. In addition, caregivers can have a role in managing medications for palliative care patients [40], and with training and support, can administer subcutaneous injections to relieve symptoms [41]. GP’s also play a role in supporting the carer, and as a result the Carer Supports Needs Assessment Tool is often integrated into palliative care within the Primary Health Care setting [42]. This tool facilitates support for family members and carers of adults with life-limiting conditions. In addition to indicating additional support they need, caregivers can also use this tool to indicate they need support for their own health and well-being in caring for a palliative care patient at home [42].

### Pharmacists

For older populations, Pharmacists can provide comprehensive health care at the end of life [43]. The Pharmacist plays a key role in ensuring that palliative care patients receive the best possible medication management [44], with community pharmacies providing medication for managing symptoms of the terminal phase [45]. Medication management for long term palliative care occurs as an interdisciplinary collaboration between pharmacists, GP’s, carers and other health professionals [46, 47], with improved management occurring when pharmacists are engaged in anticipation of the terminal phase [45, 48, 49]. Tait and Cheung [50] identified six key themes to improve community access to terminal phase medicines in Australia: medication supply, education and training, caregiver burden, safety, funding and clinical governance.

### Nurses

There are several well-defined nursing roles undertaking palliative care within the primary health setting. With adequate training and support, General Practice Nurses are able to initiate and facilitate advanced care planning conversations with patients [51]. In those cases where access to specialists is difficult, Advance Nurses provide specialized care [52]. With their knowledge of rural health resources available to patients, District Nurses are an expert advocate for implementing a person-centred goal plan for rural palliative patients [53]. Nurse practitioner coordinated palliative care appears to enable more integrated care which extends the potential for collaborative primary care and may be effective in reducing hospitalisations [54, 55]. Providing physical care to patients, managing symptoms and educating families are often roles undertaken by Specialist Nurses [56].

### Allied Health Professionals

To a lesser extent, the review has also highlighted the importance of specific allied health professionals in the provision of physical care (optimising function and nonpharmacological symptom management), social, emotional, and spiritual care [57] of palliative care patients within the primary health setting. Having Advanced Care Planner Facilitators within general practices can positively impact end-of-life care experience by referring patients to supportive programs and facilitating advance care planning [58].

## Discussion

This scoping review has identified a diverse range of individual health professional roles who work as a multidisciplinary team to meet the palliative care needs of people with life limiting illness within the Primary Health Care setting within Australia. The review has also identified that health professionals not only have a relationship to people with life-limiting illness but also their carers. While carers are not health professionals they have an important role to play in providing care for people with life limiting illness, and therefore they are often included as part of the multidisciplinary team. Carers enable care within the community through providing basic nursing, help with daily activities and with training can provide medication management and subcutaneous injections [39, 41].

The review has highlighted the pivotal role that GPs play as the patients first contact at various points along the care trajectory as a natural extension of primary care [20]. As GPs provide first-line services [59], and play an important role in engaging the multidisciplinary team of health professionals, including community services, allied health professionals, and palliative care physicians [20]. With the composition of the multidisciplinary team being determined by the patients’ palliative care needs and their accessibility to services. This multidisciplinary teamwork approach to address individual palliative care needs can be clearly seen in the work by Saurman et. al. 2019 [60] who mapped the delivery of palliative care in the Far West Local Health District in New South Wales, Australia. The Far West Local Health District network mapping has also highlighted the integration of services and care practice, identifying palliative care activities that can be undertaken by a number of health professional roles. Mapping palliative care pathways within the Primary Health Care setting provides examples to other health professionals new to palliative care and shows that policy makers and systems are currently active in this setting. The identified pathways provide Primary Health Care Networks in Australia with clear evidence on the health professionals, services and models of care that are required to meet palliative care needs within their regions.

The increase in the number of people preferring to undertake palliative care at home and the provision of financial incentives from the Australian Government through the Greater Choice for at Home Palliative Care Measure has provided momentum for increased positive end-of-life services for people with life limiting illness and their carers within the Primary Health Care setting within Australia [61]. However, there are currently obstacles to support people at home to have their symptoms well managed and to provide personal, social, and psychological support. Palliative care delivery in the Primary Health Care setting is currently challenged by meeting the needs of the increasing number of people requiring palliative care, the need to increase the workforce capacity and capability of health professionals [59]. The review findings suggest that for best practice palliative care to occur within this setting, there is a need to support those that actively provide palliative care with training, evidence to inform practice and tools.

The identification of these health professional roles and the palliative care activities that they undertake has provided the scope for developing an evidence base for continuing professional development and for clinical decision making to support palliative care within the Primary Health Care setting. The multidisciplinary team approach that has been acknowledged within several articles further extends the scope of evidence that is required to support health professionals working within the Primary Health Care setting to include referral triggers and pathways between not only health professionals but settings of care including primary care, aged care and acute care.

### Strengths and Limitations

Our scoping review benefits from several strengths. Firstly, the scoping literature review search was undertaken by a professional health librarian. The study selection process was verified by a second reviewer to ensure the inclusion and exclusion criteria were applied accurately. The scoping literature review has been a useful way to synthesise a broad range of research evidence about palliative care within the Primary Health Care setting within Australia. However, this scoping literature review would have benefitted from a search of grey literature to yield insights into broader aspects of palliative care within the Primary Health Care setting.

A limitation of this review is that the literature surveyed was limited to Australia only. However, this provides a good national example of palliative care within the Primary Health Care setting which could be adopted by other countries.

### Conclusion

This scoping review has identified health professional roles and their scope of practice in providing palliative care within the Primary Health Care setting within Australia. It has also provided examples of models of care which support people with life-limiting illness engage with other services and health professionals within the Primary Heath Care setting and across other health care settings.
