# Supplementary material for: Palliative Care Within the Primary Health Care Setting in Australia: A Scoping Review
Source: Public Health Rev. 2022 Sep 6;43:1604856. doi: 10.3389/phrs.2022.1604856 (PMC9485459; doi:10.3389/phrs.2022.1604856)
Supplement: Supplementary file 1 [file Table1.DOCX]

**Appendix 1. Database Search Filters** (Australia, 2022).

Database(s): **Ovid MEDLINE(R) and Epub Ahead of Print, In-Process & Other Non-Indexed Citations, Daily and Versions(R)**1946 to November 25, 2020
Search Strategy:

| **#** | **Searches** | **Results** |
| --- | --- | --- |
| 1 | (advance care plan* or attitude to death or bereavement or terminal care or supportive care or terminally ill or terminal illness or palliat* or hospice* or end of life or advance directive* or death attitude* or terminal cancer or supportive care or "death and dying").mp. [mp=title, abstract, original title, name of substance word, subject heading word, floating sub-heading word, keyword heading word, organism supplementary concept word, protocol supplementary concept word, rare disease supplementary concept word, unique identifier, synonyms] | 172772 |
| 2 | Primary Health Care/ or allied health occupations/ or audiology/ or occupational therapy/ or physical therapy specialty/ or speech-language pathology/ | 99856 |
| 3 | General Practitioners/ or allied health personnel/ or community health workers/ or home health aides/ or licensed practical nurses/ or audiologists/ or nutritionists/ or occupational therapists/ or physical therapists/ | 29435 |
| 4 | (general practi* or family practi* or gp* or family physician* or family doctor* or primary care or primary health).mp. | 465523 |
| 5 | health professional*.mp. | 56407 |
| 6 | (social worker* or speech patholog* or dietit* or dietic* or physio* or psychologist* or therapist* or home care).mp. | 5559360 |
| 7 | (audiolog* or nutritionist*).mp. | 15694 |
| 8 | (allied health or community or home care or care worker*).mp. | 650242 |
| 9 | (home based or home health or health visit* or home visit).mp. | 24013 |
| 10 | (social worker* or speech patholog* or dietit* or dietic* or physio* or psychologist* or therapist* or nurse*).mp. | 5849832 |
| 11 | 2 or 3 or 4 or 5 or 6 or 7 or 8 or 9 or 10 | 6796468 |
| 12 | (australia* or northern territory or tasmania* or new south wales or victoria* or queensland*).mp,in,jn | 795586 |
| 13 | 1 and 11 and 12 | 2695 |
| 14 | limit 13 to yr="2015 -Current" | 1330 |

PubMed

((advance care plan*[tiab] OR attitude to death[tiab] OR bereavement[tiab] OR terminal care[tiab] OR supportive care[tiab] OR terminally ill[tiab] OR terminal illness[tiab] OR palliat*[tiab] OR hospice*[tiab] OR end of life[tiab] OR advance directive*[tiab] OR death attitude*[tiab] OR terminal cancer[tiab] OR supportive care[tiab]) AND (general practi*[tiab] OR family practi*[tiab] OR gp*[tiab] OR family physician*[tiab] OR family doctor*[tiab] OR primary care[tiab] OR primary health[tiab] OR home based[tiab] OR home health[tiab] OR health visit*[tiab] OR home visit[tiab] OR social worker*[tiab] OR speech patholog*[tiab] OR dietit*[tiab] OR dietic*[tiab] OR physio*[tiab] OR psychologist*[tiab] OR therapist*[tiab] OR nurse*[tiab] OR allied health[tiab] OR community[tiab] OR home care[tiab] OR care worker*[tiab] OR audiolog*[tiab] OR nutritionist*[tiab] OR health professional*[tiab]) AND (australia*[tiab] OR northern territory[tiab] OR tasmania*[tiab] OR new south wales[tiab] OR victoria*[tiab] OR queensland*[tiab])) NOT medline[sb]

Scopus

( ( TITLE ( "general practi*" OR "family practi*" OR gp* OR "family physician*" OR "family doctor*" OR "primary care" OR "primary health" OR "health professional*" ) OR ABS ( "general practi*" OR "family practi*" OR gp* OR "family physician*" OR "family doctor*" OR "primary care" OR "primary health" OR "health professional*" ) OR TITLE ( "social worker*" OR "speech patholog*" OR dietit* OR dietic* OR physio* OR psychologist* OR therapist* OR audiolog* OR nutritionist* OR nurse* OR "allied health" OR community OR "home care" OR "care worker*" OR "home based" OR "home health" OR "health visi) OR ABS(" social AND worker* " OR " speech AND patholog* " OR dietit* OR dietic* OR physio* OR psychologist* OR therapist* OR audiolog* OR nutritionist* OR nurse* OR " allied AND health " OR community OR " home AND care " OR " care AND worker* " OR " home AND based " OR " home AND health " OR " health AND visi ) ) ) AND ( ( TITLE ( "advance care plan*" OR "attitude to death" OR bereavement OR "terminal care" OR "supportive care" OR "terminally ill" OR "terminal illness" OR palliative OR hospice* OR "end of life" OR "advance directive*" OR "death attitude*" OR "terminal cancer" ) OR ABS ( "advance care plan*" OR "attitude to death" OR bereavement OR "terminal care" OR "supportive care" OR "terminally ill" OR "terminal illness" OR palliative OR hospice* OR "end of life" OR "advance directive*" OR "death attitude*" OR "terminal cancer" ) ) ) AND ( ( TITLE ( australia* OR "northern territory" OR tasmania* OR "new south wales" OR queensland* OR victoria ) OR ABS ( australia* OR "northern territory" OR tasmania* OR "new south wales" OR queensland* OR victoria ) ) ) AND ( LIMIT-TO ( AFFILCOUNTRY , "Australia" ) ) AND ( LIMIT-TO ( PUBYEAR , 2020 ) OR LIMIT-TO ( PUBYEAR , 2019 ) OR LIMIT-TO ( PUBYEAR , 2018 ) OR LIMIT-TO ( PUBYEAR , 2017 ) OR LIMIT-TO ( PUBYEAR , 2016 ) OR LIMIT-TO ( PUBYEAR , 2015 ) OR LIMIT-TO ( PUBYEAR , 2014 ) OR LIMIT-TO ( PUBYEAR , 2013 ) OR LIMIT-TO ( PUBYEAR , 2012 ) OR LIMIT-TO ( PUBYEAR , 2011 ) OR LIMIT-TO ( PUBYEAR , 2010 ) OR LIMIT-TO ( PUBYEAR , 2009 ) OR LIMIT-TO ( PUBYEAR , 2008 ) OR LIMIT-TO ( PUBYEAR , 2007 ) OR LIMIT-TO ( PUBYEAR , 2006 ) OR LIMIT-TO ( PUBYEAR , 2005 ) OR LIMIT-TO ( PUBYEAR , 2004 ) OR LIMIT-TO ( PUBYEAR , 2003 ) OR LIMIT-TO ( PUBYEAR , 2002 ) OR LIMIT-TO ( PUBYEAR , 2001 ) OR LIMIT-TO ( PUBYEAR , 2000 ) ) AND ( LIMIT-TO ( DOCTYPE , "ar" ) OR LIMIT-TO ( DOCTYPE , "re" ) ) AND ( LIMIT-TO ( LANGUAGE , "English" ) )

ProQuest

Articles in Scholarly Journals

(ti("general practi*" OR "family practi*" OR gp* OR "family physician*" OR "family doctor*" OR "primary care" OR "primary health" OR "health professional*") OR ab("general practi*" OR "family practi*" OR gp* OR "family physician*" OR "family doctor*" OR "primary care" OR "primary health" OR "health professional*") OR ti("social worker*" OR "speech patholog*" OR dietit* OR dietic* OR physio* OR psychologist* OR therapist* OR audiolog* OR nutritionist* OR nurse* OR "allied health" OR community OR "home care" OR "care worker*" OR "home based" OR "home health" OR "health visit*" OR "home visit*") OR ab("social worker*" OR "speech patholog*" OR dietit* OR dietic* OR physio* OR psychologist* OR therapist* OR audiolog* OR nutritionist* OR nurse* OR "allied health" OR community OR "home care" OR "care worker*" OR "home based" OR "home health" OR "health visit*" OR "home visit*")) AND (ti("advance care plan*" OR "attitude to death" OR bereavement OR "terminal care" OR "supportive care" OR "terminally ill" OR "terminal illness" OR palliative OR hospice* OR "end of life" OR "advance directive*" OR "death attitude*" OR "terminal cancer") OR ab("advance care plan*" OR "attitude to death" OR bereavement OR "terminal care" OR "supportive care" OR "terminally ill" OR "terminal illness" OR palliative OR hospice* OR "end of life" OR "advance directive*" OR "death attitude*" OR "terminal cancer")) AND (ti(australia* OR "northern territory" OR tasmania* OR "new south wales" OR queensland* OR Victoria) OR ab(australia* OR "northern territory" OR tasmania* OR "new south wales" OR queensland* OR Victoria))

| Cinahl (Ebsco) | Monday, November 30, 2020 9:11:55 PM |
| --- | --- |

| **#** | **Query** | **Results** |
| --- | --- | --- |
| S1 | (MH "Primary Health Care") | 64,595 |
| S2 | (MH "Physicians, Family") | 20,222 |
| S3 | (MH "Community Health Nursing") OR (MH "Community Health Services") OR (MH "Community Health Workers") | 50,622 |
| S4 | (MH "Home Health Aides") | 1,362 |
| S5 | (MH "Allied Health Professions") OR (MH "Audiology") | 7,124 |
| S6 | (MH "Occupational Therapists") OR (MH "Physical Therapists") OR (MH "Speech-Language Pathologists") OR (MH "Social Workers") OR (MH "Dietitians") OR (MH "Audiologists") | 44,747 |
| S7 | “general practi*” or “family practi*” or gp* or “family physician*” or “family doctor*” or “primary care” or “primary health” or “health professional*” | 205,339 |
| S8 | “social worker*” or “speech patholog*” or dietit* or dietic* or physio* or psychologist* or therapist* or audiolog* or nutritionist* or nurse* | 1,311,018 |
| S9 | “allied health” or community or “home care” or “care worker*” or “home based” or “home health” or “health visit*” or “home visit*” | 382,751 |
| S10 | S1 OR S2 OR S3 OR S4 OR S5 OR S6 OR S7 OR S8 OR S9 | 1,759,805 |
| S11 | “advance care plan*” or “attitude to death” or bereavement or “terminal care” or “supportive care” or “terminally ill” or “terminal illness” or palliat* or hospice* or “end of life” or “advance directive*” or “death attitude*” or “terminal cancer” or “supportive care” or "death and dying" | 112,684 |
| S12 | AF ( australia* or “northern territory” or tasmania* or “new south wales” or queensland* ) OR TI ( australia* or “northern territory” or tasmania* or “new south wales” or queensland* or victoria ) OR AB ( australia* or “northern territory” or tasmania* or “new south wales” or queensland* or victoria ) | 244,539 |
| S13 | S10 AND S11 AND S12 | 2,019 |
| S14 | S10 AND S11 AND S12 2015-Current | 953 |
