# Supplementary material for: Palliative Care Within the Primary Health Care Setting in Australia: A Scoping Review
Source: Public Health Rev. 2022 Sep 6;43:1604856. doi: 10.3389/phrs.2022.1604856 (PMC9485459; doi:10.3389/phrs.2022.1604856)
Supplement: Supplementary file 2 [file Table2.DOCX]

**Appendix 2. Characteristics of Sources of Evidence** (Australia, 2022).

| **Author, Year** | **Study Design** | **Population** | **Workforce** | **Main Finding** |
| --- | --- | --- | --- | --- |
| Antoine et. al. 2020 ([48](#_ENREF_48)) | Qualitative research | Cancer patients | Community Pharmacies | “Ascertaining individual tipping points based on motivators or enablers and barriers with subsequent tailoring of supportive care programs will be more likely to meet patients’ individual needs and may increase the likelihood of delivering appropriate care via community pharmacies.” Antonine et. al. 2020 p.1 ([48](#_ENREF_48)) |
| Aoun et. al. 2015 ([41](#_ENREF_41)) | One part of a three-part evaluation of a stepped wedge cluster trial | Family Care Givers | Family Care Givers | “Integrating the Carer Support Needs Assessment Tool in existing practice is fundamental to achieving better caregiver outcomes.” Aoun et. al. 2015 p.929 ([41](#_ENREF_41)) |
| Aoun et. al 2016 ([61](#_ENREF_61)) | Questionnaire | Nurses who had referred clients to the Home Alone Models of Care Project | Nurses | “The data demonstrated that concern for the clients’ wellbeing guided the clinical input that the nurses provided to all clients.  However, the nurses recognised that the models of care might not translate into granting clients their wishes regarding places of terminal care and death.” Aoun et. al 2016 p.17 ([61](#_ENREF_61)) |
| Ash et. al. 2016 ([62](#_ENREF_62)) | General information piece | Clinical Workforce | Nurses | “All nurses thus require some level of knowledge and skills in how to apply the principles of palliative care.  A National Palliative Care Workforce Development Framework has been developed to provide guidance for individual healthcare providers, education providers, health service managers and policy makers to ensure all healthcare providers are equipped with capabilities relevant to their context of practice.” Ash et. al. 2016 p.31 ([62](#_ENREF_62)) |
| Bernardes et. al. 2019  ([63](#_ENREF_63)) | Data from two cross‐sectional supportive care needs studies were matched. Descriptive statistics were used to compare type and prevalence of 24 need items. | Australian adults diagnosed with cancer | Patient | “Variations in the unmet supportive care needs between Indigenous and Non‐Indigenous people with cancer may guide health professionals to target specific needs when preparing care plans.” Bernardes et. al. 2019 p.1 ([63](#_ENREF_63)) |
| Bernardes et. al. 2018 ([64](#_ENREF_64)) | Participants were assessed through a face- to- face interview | Indigenous cancer patients | Indigenous Patient Navigator | “Indigenous patient navigator programmes show promise in addressing barriers to cancer care and facilitation of patient self-efficacy.” Bernardes et. al. 2018 p.1 ([64](#_ENREF_64)) |
| Borthwick et. al. 2020  ([65](#_ENREF_65)) | Four interdisciplinary focus groups and two in-depth interviews with participants were conducted and thematically analysed using an interpretivist inquiry paradigm. | Individuals living with a chronic condition | Allied Health Professionals | “The data suggest that advance care planning by allied health professionals in the primary care setting is limited.” Borthwick et. al. p.383 ([65](#_ENREF_65)) |
| Carey et. al. 2019 ([66](#_ENREF_66)) | Systematic review of quantitative data-based articles was conducted. | Primary Care Practitioners | Primary Care Practitioners | “A lack of confidence was reported across many care areas including treatments, symptom management, and psychological and communication issues, reflecting the holistic nature of palliative care.” Carey et. al. 2019 p. 1142 ([66](#_ENREF_66)) |
| Carey et. al. 2017 ([67](#_ENREF_67)) | A pre and post design was used to compare changes in Emergency Department (ED) attendances and hospital admissions following the commencement of the service | Patients attending the respite service including  Aboriginal and Torres Strait Islander people | Respite service staff | “The respite facility filled a gap in services for those marginalised Aboriginal and Torres Strait Islanders people suffering from chronic and complex conditions who are not yet in need of intensive end of life care.” Carey et. al. 2017 p.4 ([67](#_ENREF_67)) |
| Carey et. al. 2016 ([68](#_ENREF_68)) | Semi-structured interviews were conducted with patients, carers, referrers, and stakeholders. Interpretative phenomenological analysis was used inductively to analyse the transcripts. | Patients, Carers, Referrers, and Stakeholders | Day respite service staff | “The findings clearly indicate an improvement in quality of life for respite patients and their carers. The respite service enabled improved care coordination of chronic and complex patients as well as improved medication compliance and symptom management.” Carey et. al. 2016 p.1 ([68](#_ENREF_68)) |
| Carmont et. al. 2017 ([25](#_ENREF_25)) | Systematic review which used a narrative framework to describe the findings. | General Practitioners | General Practitioner | “There is some evidence that integrated palliative care can reduce hospitalisations and maintain functional status.” Carmont et. al. 2017 p.385 ([25](#_ENREF_25)) |
| Champion 2015 ([69](#_ENREF_69)) | A mixed method approach was chosen for the study using descriptive analysis of routinely collected client demographic and clinical data, and conventional content analysis of case note entries | Deceased adults registered with the Northern Yorke Peninsula and Port Pirie Palliative Care Services with a recorded preferred site of death of home and who died in hospital. | Patient | “Services can now consider these findings and develop local strategies to improve support for clients with a preference to die at home, and in particular, those that focus on improving caregiver training and information.” Champion 2015 p.6 ([69](#_ENREF_69)) |
| Clark et. al. 2016  ([70](#_ENREF_70)) | Using prospectively collected data from participating palliative care services in the Australian Palliative Care Outcomes Collaboration between July 1, 2013, and December 31, 2014, factors associated with worse symptom experiences were explored using logistic regression modeling. | People identified as being in the terminal phase of their life-limiting illness. | Palliative Care Specialist | “It is commonly stated that people want to die at home, but achieving this requires families and friends to be willing caregivers able to mobilize sufficient support of health professionals, including a family physician. As death approaches, some will change their minds regarding the place of care, preferring to move from a community setting to an inpatient setting.” Clark et. al. 2016 p. 1292 ([70](#_ENREF_70)) |
| Collier et. al. 2019 ([71](#_ENREF_71)) | Participatory qualitative study.  The methodology of positive organisational scholarship in healthcare was combined with video-reflexive ethnography. | Patients, families, Clinicians | Clinicians | “These findings reflect a meta-synthesis of the critical components of home-based palliative care from the perspective of patients and carers. These were found to include: security – that is, on-call availability and home visits; competence – effective symptom control, and skilful communication. The findings support those of a recent investigation of 11 specialist home-based palliative care services in Canada, whereby intra-team communication and team-building were viewed as more important than: clinical tools; standardised processes; specialised expertise; timeliness; physical symptom; psychosocial support; spiritual management; education; peace and fulfilment; as well as patient advocacy. Interprofessional teams that care for people with high-level needs and operate in complex situations need to adapt to rapidly changing environments. The findings from this study suggest that home-based palliative care teams do this by engaging with systems and processes to make them work.” Collier et. al. 2019 p.99 ([71](#_ENREF_71)) |
| Collier et. al. 2015 ([72](#_ENREF_72)) | Randomised controlled plot of patient/GP dyads | General practitioners and patients | General Practitioners | “Results illustrate the potential of targeted academic detailing by a specialist consultant to GPs, enhancing their knowledge of diagnosis of reversible causes and management and confidence in dealing with symptomatic breathlessness for specific patients. Building the capacity of the existing workforce to adopt new evidence as it emerges using innovative and cost-effective strategies will be critical.” Collier et. al. 2015 p.4 ([72](#_ENREF_72)) |
| Craswell et. al. 2020 ([54](#_ENREF_54)) | Mixed methods | Acutely ill older adults living in aged care facilities. | Nurse Practitioner | “This study provides emerging evidence that while progressing towards endorsement, a nurse practitioner candidate can contribute to the delivery of primary care required for this cohort.” Craswell et. al. 2020 p. 286 ([54](#_ENREF_54)) |
| Cross et. al. 2020 ([46](#_ENREF_46)) | Focus groups | Patients, General Practitioners, Nurses and Pharmacists | Patients, General Practitioners, Nurses and Pharmacists | “The role of the carer in medication management was described as fundamental by all stakeholders.The patient’s GP was seen as the main care coordinator for a person living with dementia, with interdisciplinary input essential for optimising patient-centred care.” Cross et. al. 2020 p.6 ([46](#_ENREF_46)) |
| Cross, Fischer et. al. 2020 ([73](#_ENREF_73)) | Single-centre, prospective, comparative cohort study comprised a clinical chart audit and a survey of patient and carer experience. | Patients referred and admitted to the Community Specialist Palliative Care Service. | Clinical Nurses (Case Coordinators), doctors and counsellors | “The important additions to the standard community palliative care service included case management by a dedicated doctor–nurse team, increased allied health access and utilisation, and availability of an ‘Intensive Specialist Palliative Care’ component if needed, including AIN support at all hours.  This study suggests that models that offer personalised care, enhance patient choice, provide increased carer support, and allow flexibility to transition between locations of care, are likely to be the key to improving end-of-life experiences for patients and families, and to address rising health care costs.” Cross, Fischer et. al. 2020 p.935 ([73](#_ENREF_73)) |
| Deckx et. al. 2019 ([32](#_ENREF_32)) | A qualitative study of 15 Australian GPs using semi-structured interviews, examining end-of-life care of one of their randomly selected, deceased patients. Interviews were analysed using a general inductive approach. | General Practitioners | General Practitioners | “GPs were aware of their patients’ approaching end of life and care was adjusted accordingly.” Deckx et. al. 2019 p.1 ([32](#_ENREF_32)) |
| Deckx et. al. 2020 ([30](#_ENREF_30)) | Semi-structured interviews. Transcripts were analysed using thematic analysis. | General Practitioners | General Practitioners | “GPs described end-of-life conversations as a process. This involved initially preparing the ground for the discussion by establishing strong doctor–patient relationships; gauging patients’ readiness to engage; and managing time availability. Entry points to the conversation varied and could include responding to patient initiation, incorporation into routine care, raising discussions around prognosis or less directly discussing hypothetical deterioration or enquiring about patients’ views about death.” Deckx et. al. 2020 p. 404 ([30](#_ENREF_30)) |
| Ding et. al. 2018 ([74](#_ENREF_74)) | Systematic Review | General Practitioner | General Practitioner | “One major issue related to the inequity of palliative care among patients with different illnesses. Patients diagnosed with cancer often received a higher level of palliative care, and their families were more fully involved in such care. Other specific areas of concern included the infrequent use of validated scales in symptoms assessment, suboptimal symptom relief, and limited support in social and spiritual aspects of EOL. Amongst some groups of doctors, there was limited awareness of patient’s preferences for care and preferred place of death. Limited resource availability and lack of palliative care training opportunities were also reported as areas of concern.” Ding et. al. 2018 p.1801 ([74](#_ENREF_74)) |
| Ding et. al. 2019 ([33](#_ENREF_33)) | A qualitative study involving five focus groups. | State registered Medical Practitioners with approved general practice qualifications who care for patients across all age groups and clinical conditions and other stakeholders whose work was relevant to palliative and end-of-life care including Policy Makers, Palliative Care Specialists and Palliative Care Researchers. | General Practitioners | “The rural GPs’ central role in end-of-life care was recognized by the majority of participants. Inappropriate payment models discouraged GPs’ involvement in some aspects of end-of-life care, such as case conferences and home visits. Compared to GPs in urban settings, those in rural/regional communities often reported closer doctor-patient relationships and better care integration and collaboration.” Ding et. al. 2019 p.1 ([33](#_ENREF_33)) |
| Disalvo et. al. 2019 ([45](#_ENREF_45)) | Semi-structured interviews were undertaken via telephone and face-to-face. | Accredited Pharmacists | Pharmacists | “This research demonstrates the importance of interdisciplinary collaboration between pharmacists, GPs and other health professionals to improve medication management for long term care residents with advanced dementia.” Disalvo et. al. 2019 p.960 ([45](#_ENREF_45)) |
| Dong et. al. 2016 ([75](#_ENREF_75)) | Semi-structured interviews. Transcripts were analysed using thematic analysis and adapted grounded theory. | Palliative Care Physicians, Oncologists, General Practitioners, Nurses, Allied Health Professionals | Palliative Care Physicians, Oncologists, General Practitioners, Nurses, Allied Health Professionals | “Our findings highlight the complexity of processes and components in the effective management of multiple symptoms for patients with advanced cancer, which require effective collaboration across settings. Our results suggest that clinical experience alone was insufficient as clinicians depended on team work and knowledge exchange to instil hope for symptom relief and empowerment within the patient and family.” Dong et. al. 2016 p.714-715 ([75](#_ENREF_75)) |
| Dunning 2016 ([76](#_ENREF_76)) | Opinion piece | Diabetic patients | Palliative care clinicians | “The interdisciplinary care team and the person with diabetes and their families must be involved in care decisions and understand the rationale for and the aims of integrated palliative/usual care.” Dunning 2016. p. 306 ([76](#_ENREF_76)) |
| Edwards et. al. 2019 ([77](#_ENREF_77)) | Semi-structured and cyclical discussions with key informants alongside internal document reviews. | Nurse practitioner | Nurse Practitioner | “Home can mean different things to different people, so it is equally important to have access to quality care in the place of ones’ choosing, such as timely and affordable specialist palliative services and appropriate medications and symptom control.” Edwards et. al. 2019 p.247 ([77](#_ENREF_77)) |
| Ewald et. al. 2018 ([78](#_ENREF_78)) | Quasi-experimental design utilising an intervention and control group based on neighbouring rural Community Nursing Areas (CNA) | Patients referred by General Practices and Specialists. General Practitioners | General Practice Registrar | “This pilot provides preliminary evidence that a General Practice Registrar palliative care facilitator can significantly reduce rural palliative care patients’ hospital admissions and inpatient days.” Ewald et. al. 2018 p.2 ([78](#_ENREF_78)) |
| Fletcher et. al. 2016 ([79](#_ENREF_79)) | Focus groups | General Practitioners, General Practice Registrars, Practice Nurses, Community Nurses, Hospital Nurses | General Practitioners, General Practice Registrars, Practice Nurses, Community Nurses, Hospital Nurses | “In rural communities some HCPs, particularly GPs, work across different healthcare settings. Some GPs working in rural towns suggested that ACP discussions do not need to be documented because, based on past discussions, GPs know what care their patients would want, and would typically provide care for their patients in primary care and local hospital settings.” Fletcher et. al. 2016 p. 425 ([79](#_ENREF_79)) |
| Halkett et. al. 2015 ([21](#_ENREF_21)) | Qualitative research. Semi-structured interviews. Analysed using a constant comparative methodology. | Participants were eligible if they were diagnosed with advanced cancer and referred for palliative radiotherapy. | General practitioner | “Patients want GPs to have varying levels of involvement following an advanced cancer diagnosis. Patients wished to maintain continuity of care for their non-cancer related issues and healthcare of their family members.” Halkett et. al. 2015 p.662 ([21](#_ENREF_21)) |
| Harvey et. al. 2019 ([80](#_ENREF_80)) | Qualitative study involving thematic analysis of interview data. | Staff members of local health and support services. | Health Service Professionals | “Rural and regional practitioners need to be aware of the effect long‐term continuity of care from health practitioners and connections with health services has on advance care plan creation, and whether the paucity of written Plans effects end‐of‐life care.” Harvey et. al. 2019 p.563 ([80](#_ENREF_80)) |
| Healy et. al. 2018 ([40](#_ENREF_40)) | Prospective randomized controlled trial | Laycarers who had volunteered to prepare and administer subcutaneous injections were targeted. | Laycarers | “Upskilled laycarers can confidently administer subcutaneous injections for loved ones, regardless of who prepares injections. This can improve patient outcomes and potentially decrease unwanted admissions to impatient facilities.” Healy et. al. 2018 p. 1208 ([40](#_ENREF_40)) |
| Hermann et. al. 2019 ([19](#_ENREF_19)) | Qualitative interview study. Semi-structured telephone interviews. Data were analysed using qualitative content analysis. | General Practitioners | General Practitioners | “Best practice palliative care was perceived to be proactive and responsive to a wide range of patient and family needs. Many participants indicated a need for relational continuity, which involves GPs establishing a care pathway from diagnosis to palliation, coordinating care across the pathway, and collaborating with other healthcare providers. A number of participants perceived palliative care as a natural extension of primary care and indicated that best practice palliative care mainly requires experiential knowledge and good communication skills, rather than specialised medical knowledge.” Hermann et. al. 2019 p.2 ([19](#_ENREF_19)) |
| Holloway et. al. 2015 ([81](#_ENREF_81)) | A descriptive, exploratory qualitative design was used. Sampling was purposive. Data were collected during audiotaped, semi-structured, individual and focus group interviews. Thematic analysis was undertaken. | Nurses, Managers, Consumers, Psychosocial Support Workers, Allied Health Personnel, Medical Practitioners, Aboriginal Health Workers, Care Workers, Researchers or Educators, Volunteer Support Workers, Pastoral Care Workers or Chaplains | Nurses, Managers, Consumers, Psychosocial Support Workers, Allied Health Personnel, Medical Practitioners, Aboriginal Health Workers, Care Workers, Researchers or Educators, Volunteer Support Workers, Pastoral Care Workers or Chaplains | “The guidelines needed to tailor the provision of high-quality community aged care in which the older person’s condition is life limiting, recognising that predicting life expectancy can be problematic (e.g. in dementia). A key feature of the findings of this study is the recognition of the significant role that family carers have in the provision of care in the community setting.” Holloway et. al. 2015 p.25 ([81](#_ENREF_81)) |
| Hudson et. al. 2015 ([82](#_ENREF_82)) | A randomised controlled trial. | Caregivers | Caregivers | “We have shown that a tailored psychoeducational intervention aimed at improving information and promoting well-being had some protective effect against the increase in distress associated with the death of a relative.” Hudson et. al. 2015 p.23 ([82](#_ENREF_82)) |
| Jiang et. al. 2020 ([83](#_ENREF_83)) | Prospective mixed-methods pilot study | Newly engaged adult patients and their caregivers of a community palliative-care service | Palliative-care Physicians | “Although not a stand-alone solution, our study supports telehealth as being a feasible method of extending specialist palliative-care support to rural communities in an integrated fashion. The solution was delivered simply by a generic laptop with little set-up time, and was able to integrate into standard community palliative-care nursing visits.” Jiang et. al. 2020 p.6-7 ([83](#_ENREF_83)) |
| Johns et. al. 2019 ([84](#_ENREF_84)) | Qualitative interviews | Social Workers, Community Health Nurses, Community Workers, Palliative Clients / Carers | Social Workers, Community Health Nurses, Community Workers, Palliative Clients / Carers | “This study highlights the roles of social workers working with palliative clients in rural and remote Queensland.” Johns et. al. 2019 p.6 ([84](#_ENREF_84)) |
| Johnson et. al. 2018 ([17](#_ENREF_17)) | Systematic review | General practitioners, practice nurse | General Practitioners, Practice Nurse | “With limited specialist PC resources available in the community, it is often GPs who provide and co-ordinate EoLC in collaboration with community-based support services.  The increasing burden of multimorbidity provides a natural role for practice nurses in identifying people at risk of dying.” Johnson et. al. 2018 p. ([17](#_ENREF_17)) |
| Johnson et. al. 2020 ([23](#_ENREF_23)) | In-depth, semi-structured, telephone interviews | People diagnosed with cancer, their Informal Carers and General Practitioners | Informal Carers, General Practitioners | “In this study, we identified essential characteristics of general practice that were perceived by patients and carers to be essential for optimal EOL care.  Comprehensive EOL care that meets the needs of people dying with cancer is within the resources and scope of Australian rural and regional GPs and communities.  Optimally, this care included skilful management of physical symptoms as well as psychological support, early intervention when concerns were either anticipated or identified and the access to, and co-ordination of, social and interdisciplinary support.” Johnson et. al. 2020 p.2161 ([23](#_ENREF_23)) |
| Khalil et. al. 2019 ([51](#_ENREF_51)) | Online survey | Nurses | Nurses | “Advanced nurses’ roles have the opportunity to provide specialized care where access to specialist physicians is challenging.” Khalil et. al. 2019 p.297 ([51](#_ENREF_51)) |
| Kirby et. al. 2016 ([85](#_ENREF_85)) | Narrative synthesis was adopted to synthesise findings from qualitative and quantitative studies. | Urban and rural terminally ill patients living alone | Caregivers | “Important were as follows: maintaining independence important; home help and respite care; support to get out and about; maintaining capacity for autonomy and choice and the need for dignity.” Kirby et. al. 2016 p. 296 ([85](#_ENREF_85)) |
| Kuruvilla et. al. 2018 ([43](#_ENREF_43)) | A qualitative focus group study | Wide range of consumers and health professionals with experience in palliative care | Specialist Palliative Care Pharmacist | “Policymakers and services responsible for improving palliative care services should not overlook the important role pharmacists can play in optimising medication management for these patients.” Kuruvilla et. al. 2018 p.1370 ([43](#_ENREF_43)) |
| Le et. al. 2017 ([27](#_ENREF_27)) | Survey | General Practitioners involved in palliative management of at least one cancer patient | General Practitioner | “GPs are an integral component of this support framework.  One proposed strategy to optimise the provision of quality community-based palliative care has been the development of collaborative, integrative models incorporating generalist and specialist palliative care providers”. Le et. al. 2017 p.52- 53 ([27](#_ENREF_27)) |
| Leonard et. al. 2020 ([86](#_ENREF_86)) | Network mapping and qualitative data | Networks of people who had cared for someone who had died at home in the past 3 years. | Carer | “Opportunities arose in EOL caring to build networks of support among family, friends, community and service providers.” Leonard et. al. 2020 p.6 ([86](#_ENREF_86)) |
| Littlewood et. al. 2019 ([34](#_ENREF_34)) | Qualitative descriptive methodology, involving semi‐structured face‐to‐face and telephone interviews. Thematic analysis of interview transcripts | General Practice Registrars and recently fellowed General Practitioners | General Practice Registrars and recently fellowed General Practitioners | “Given that rural GPs face a number of barriers to providing routine health care, these results highlight an important need to provide GPs and rural communities with support, education, incentive, better administrative tools, options and greater awareness of advance care planning.” Littlewood et. al. 2019 p.398 ([34](#_ENREF_34)) |
| Long 2019 ([35](#_ENREF_35)) | Editorial | Paramedic | Paramedic | “Paramedics are important stakeholders in the delivery of community-based palliative care and have been a notable omission in health service policy and planning.” Long 2019 p.289 ([35](#_ENREF_35)) |
| Lord et. al. 2019 ([36](#_ENREF_36)) | Retrospective cohort study. | Adult patients | Paramedic | “Our results demonstrate that paramedics are involved in caring for patients experiencing a health problem that may be associated with palliative care. There is evidence that ambulance services in Australia are responding to this role through the development of palliative care guidelines.” Lord et. al. 2019 p.449 ([36](#_ENREF_36)) |
| MacLeod et. al. 2015 ([38](#_ENREF_38)) | Case study | Patients | Trained Care Workers | “Care may include assistance with activities of daily living, personal and domestic care, and shopping and household tasks, providing family carers with vital support. In consultation with the specialist palliative care team and the patient’s GP, it may also include basic nursing care.” MacLeod et. al. 2015 p.28 ([38](#_ENREF_38)) |
| Mann et. al. 2017 ([57](#_ENREF_57)) | Analysis of routinely collected service-related data and questionnaire. | Patients | Advanced Care Planner Facilitators | “Co-locating Advanced Care Planner Facilitators in general practice increased the number of referrals to the program and produced higher plan completion rates.” Mann et. al. 2017 p.691 ([57](#_ENREF_57)) |
| Miller et. al. 2019 ([50](#_ENREF_50)) | Primary study | Patients | Doctors and General Practice Nurses | “With adequate training and support, General Practice Nurses are able to initiate and facilitate ACP conversations with patients.” Miller et. al. 2019 p.1 ([50](#_ENREF_50)) |
| Mills 2020 ([37](#_ENREF_37)) | Opinion piece | Paramedics | Paramedics | “Extended care paramedics have been known to respond to palliative care emergencies, effectively preventing unwanted and unnecessary hospital admissions.” Mills 2020 p.17 ([37](#_ENREF_37)) |
| Mitchell et. al. 2020 ([18](#_ENREF_18)) | Systematic literature review | General Practitioner (family physicians), General Practice Nurses | General Practitioner, General Practice Nurses | “The more comprehensive the engagement with GPs in the integrated planning and care of these patients, the better the outcome for patients, and the more likely there will be attendant economic benefits.  The usual palliative care programme assumes a predictable and mostly time limited time course.  The nature of national health systems has a major bearing on the nature of interventions that are possible.” Mitchell et. al. 2020 p.5 ([18](#_ENREF_18)) |
| Mitchell et. al. 2016 ([53](#_ENREF_53)) | Pilot of a Nurse practitioner -led, General practitioner supported care provision. | Nurse Practitioner, General Practitioner, patient | Nurse Practitioner, General Practitioner | “Nurse practitioner coordinated palliative care appears to enable more integrated care and may be effective in reducing hospitalisations.” Mitchell et. al. 2016 p.1 ([53](#_ENREF_53)) |
| Mitchell et. al. 2018 ([22](#_ENREF_22)) | Systematic review | General Practice Nurses, General Practitioners | General Practice Nurses, General Practitioners | “Most GPs are already practising EoLC without recognising it.” Mitchell et. al. 2018 p.417 ([22](#_ENREF_22)) |
| Morgan et. al. 2019 ([56](#_ENREF_56)) | Online survey | Allied Health Professionals:  Dietitians, Music Therapists, Occupational Therapists, Physiotherapists, Psychologists, Social Workers, Speech Pathologists | Allied Health Professionals:  Dietitians, Music Therapists, Occupational Therapists, Physiotherapists, Psychologists, Social Workers, Speech Pathologists | “Allied health clinicians have an active role in physical care (optimising function and nonpharmacological symptom management), social, emotional and spiritual care.” Morgan et. al. 2019 p.13 ([56](#_ENREF_56)) |
| Mounsey et. al. 2018 ([87](#_ENREF_87)) | Opinion piece | Non-cancer end-stage patients | General Practitioners | “The GP may be supported by a palliative care consultation or involvement of a Community Palliative Care team or residential aged care palliative care outreach worker.” Mounsey et. al. 2018 p.768 ([87](#_ENREF_87)) |
| Panozzo et. al. 2020 ([31](#_ENREF_31)) | A retrospective cohort study was conducted of patient hospital records with manual linkage to general practice records. | Patients aged 75 years and over  who died in the hospital | General Practitioners | “General practitioners commit time and resources to assist their patients to document their end-of-life care preferences.” Panozzo et. al. 2020 p.5 ([31](#_ENREF_31)) |
| Politis et. al. 2020 ([88](#_ENREF_88)) | A case-vignette based survey | General Practitioners | General Practitioners | “Many GPs are uncomfortable offering a palliative approach themselves to their patients with COPD, yet these patients are not routinely referred to specialist palliative care services despite their immense needs. This significant gap in healthcare presents a major challenge to the holistic management of patients with advanced COPD.” Politis et. al. 2020 p.6 ([88](#_ENREF_88)) |
| Poulos et. al. 2018 ([89](#_ENREF_89)) | Postal surveys and/or qualitative interviews, Quality of Death and Dying Questionnaire (QODD). | Family carers | Specialist Supportive Community Care Workers. Local health district community palliative care teams. | “Specialist Supportive Community Care Workers (CCWs) can effectively supplement the care provided by existing multidisciplinary palliative care teams.” Poulos et. al. 2018 p.e271 ([89](#_ENREF_89)) |
| Rabbetts et. al. 2020 ([55](#_ENREF_55)) | Integrated literature review | Nurses | Nurses | “Role of specialist nurses in access to resources such as equipment, medications, providing physical care of patients, managing symptoms and educating the family.” Rabbetts et. al. 2020 p.7 ([55](#_ENREF_55)) |
| Rainsford et. al. 2018 ([90](#_ENREF_90)) | Ethnography, utilising open-ended interviews, observations and field-notes | Patients | Family Caregivers,  General Practitioners, Palliative Care /  Community  Nurses, Residential Aged Care Director of Nursing. | “The role of the rural hospital in end-of-life care is unique within rural settings and differs from the role of urban hospitals.” Rainsford et. al. 2018 p.1576 ([90](#_ENREF_90)) |
| Reed et. al 2018 ([52](#_ENREF_52)) | A sequential mixed methods study | Nurses | District Nurse | “The willing and supported use of the autonomy available in the DN role to make time and become involved with people receiving EoL care is needed to advocate for person-centred goal planning.” Reed et. al 2018 p.10([52](#_ENREF_52)) |
| Reed et. al. 2015 ([91](#_ENREF_91)) | Literature review using interpretive scoping methodology | District nurse | District Nurse | “Under-resourcing, medicalisation and emotional relational burden could affect advocacy in rural areas.” Reed et. al. 2015 p.479 ([91](#_ENREF_91)) |
| Reed et. al. 2017 ([92](#_ENREF_92)) | Reflective exploration | District nurse | District Nurse | “The limitations of services to support district nurses in holistic person-centred caring have been shown to have an impact on the success of advocacy.” Reed et. al. 2017 p.163 ([92](#_ENREF_92)) |
| Reed et. al. 2018 ([93](#_ENREF_93)) | A pragmatic mixed method study. | Rural district nurse. | Rural District Nurse. | “The model represents new theory for DN practice supported by existing concepts found in the nursing advocacy literature.” Reed et. al. 2018 p.753 ([93](#_ENREF_93)) |
| Reymond et. al. 2016 ([16](#_ENREF_16)) | Development of a framework | Aged care patients | General Practitioners | “The framework of palliative care provides the opportunity to improve clinicians’ mindfulness regarding proactive management of clinical needs that typically emerge in the last year of life. It supports communication between the GP and the patient and their carers/ substitute decision-makers, thereby enabling informed management plans to be developed in accordance with the patient’s personal choices.” Reymond et. al. 2016 p.77 ([16](#_ENREF_16)) |
| Reymond et. al. 2018 ([26](#_ENREF_26)) | Synthesis of existing evidence-based frameworks | Palliative care team | General Practitioner, Nurses, Social Workers, Occupational Therapists, Allied Health Professionals, local Pharmacist, Specialist Palliative Care services,  Carers | “Palliative care is multifaceted, team-based care that sits well within the GP specialist scope of practice. It is person-centred and family-centred care, provided to a person with active, progressive advanced disease(s) who is expected to die in the short term and for whom the primary treatment goal is to optimise quality of life.  Most home-based palliative care teams are led and proactively coordinated by the GP. Team members vary and should be determined by patient needs and local availability of professionals. Members include clinicians such as nurses, social workers, occupational therapists, other allied health professionals and the local pharmacist. With more complex patients, specialist palliative care services may be enlisted to complement the skills of the local team. The carer’s role is worthy of mention.  Carers usually know the patient most intimately and are motivated to help.” Reymond et. al. 2018 p.749 ([26](#_ENREF_26)) |
| Rhee et. al. 2018 ([24](#_ENREF_24)) | Qualitative description methodology using semi-structured interviews | General Practitioners, Specialist Palliative Care Clinicians | General Practitioners, Specialist Palliative Care Clinicians | “The SPCC participants identified two key roles that GPs should play in end-of-life care: initiating end-of-life care planning with patients and making referrals to specialist palliative care services if indicated and being the primary clinician in charge of day-to-day care of patients with end-of-life care needs.” Rhee et. al. 2018 p.6 ([24](#_ENREF_24)) |
| Rosenberg 2017 ([94](#_ENREF_94)) | Opinion piece | Multidisciplinary teams | General Practitioners, Practice Managers, Practice Nurses | “While key players in primary care include GPs, practice managers, practice nurses, and community care services, these personnel are not always included in multidisciplinary teams, which can impact quality of palliative and end of life care. It is clear that integrated models of PEoLC are most effective, where GPs and their interdisciplinary clinical partners communicate openly, share care pathways, have appropriate training, and are supported by viable funding models.” Rosenberg 2017 p.39 ([94](#_ENREF_94)) |
| Saurman et. al. 2019 ([59](#_ENREF_59)) | Semi-structured interviews | Specialist palliative care service and generalist healthcare providers | Patients, families and other providers associated with palliative care – from hospital staff and Allied Health Professionals to Pharmacists and volunteers | “Everyone interviewed said that they knew their community and patients well and that they had good communication with their patients, families and other providers associated with palliative care – from hospital staff and allied health professionals to pharmacists and volunteers.” Saurman et. al. 2019 p.4 ([59](#_ENREF_59)) |
| Senior et. al. 2019 ([15](#_ENREF_15)) | Systematic literature review. | General Practitioners | General Practitioners | “The role is diverse and includes symptom management, pain management and non-malignant disease management.  A workable model to co-ordinate multidisciplinary care around the needs of PC patients and carers uses interprofessional case conferences between the GP, a specialist palliative medicine physician and other team members, and sometimes the patient and/or carer, and is conducted using communications technology.” Senior et. al. 2019 p.7 ([15](#_ENREF_15)) |
| Shahid et. al. 2018 ([95](#_ENREF_95)) | Systematic review | Indigenous patients | Patient | “Seven key principles for Indigenous palliative care service delivery: equity (equal access); autonomy/empowerment (respecting patients’ choices); trust (acknowledgement and consideration of the historical context of colonisation and its impact on the lives of Indigenous people and empathy while providing care); humane (non-judgemental care with a focus on quality of life and choice for patients and their families); seamless care (collaboration of a multidisciplinary team of health professionals and community-based organisations, working together across the continuum of care); emphasis on living (rather than on dying), and cultural respect (respect towards cultural practices and beliefs, culturally-based lifestyle).” Shahid et. al. 2018 p. 6 ([95](#_ENREF_95)) |
| Sinclair et. al. 2016 ([29](#_ENREF_29)) | Case study | Patients | General Practitioners, Professional Mediators | “The GP can develop an approach to ACP that promotes patient autonomy while remaining family-centred.” Sinclair et. al. 2016 p.71 ([29](#_ENREF_29)) |
| Sinclair, Gates et. al. 2016 ([96](#_ENREF_96)) | Factorial survey | General Practitioner | Primary Care Physicians / General Practitioner | “Primary care physicians are well placed to identify patients in need of advance care planning (ACP) and initiate ACP in advance of an acute situation.” Sinclair, Gates et. al. 2016 p.718 ([96](#_ENREF_96)) |
| Spelten et. al. 2019 ([97](#_ENREF_97)) | Semi-structured interviews | Family members and Nurses | Nurses | “This study has demonstrated the positive impact of an end‐of‐life service, while at the same time pointing to concerns of the nursing staff on the suitability of the model of care. Health care workers and communities alike need to be educated and have conversations on end‐of‐life care.” Spelten et. al. 2019 p.336 ([97](#_ENREF_97)) |
| Swerissen et. al. 2015  ([3](#_ENREF_3)) | Opinion piece | Patient | Health professionals, Carers | “Palliative care is not discussed, offered or provided, and services are variable, inconsistent and fragmented, particularly for support at home and in the community. Women, single people, older people and Indigenous people die in hospital at a higher rate than the general population. People with culturally and linguistically diverse backgrounds and those from rural communities are more likely to find access to services more difficult.” Swerissen et. al. 2015 p.10 ([3](#_ENREF_3)) |
| Tait, Chakraborty et. al. 2020 ([42](#_ENREF_42)) | Systematic review | Older persons | Pharmacists | “The multifaceted role of pharmacists presents an opportunity to provide comprehensive health care for older populations at the end of their life.” Tait, Chakraborty et. al. 2020 p.1 ([42](#_ENREF_42)) |
| Tait, Cuthbertson et. al. 2020 ([39](#_ENREF_39)) | Structured interviews | Patients | Care Givers | “The findings of this study provide insights to inform the development of palliative care service models to support informal caregivers in the management of medications for people with a life-limiting illness.” Tait, Cuthbertson et. al. 2020 p. 1084 ([39](#_ENREF_39)) |
| Tait, Horwood et. al. 2020 ([47](#_ENREF_47)) | Repeat survey | Community pharmacies | Community Pharmacists | “Palliative care organisations should raise the issue of access to medicines with community-based prescribers in advance, to ensure that their patients are adequately prepared for symptom management, where a home death is favoured.” Tait, Horwood et. al. 2020 p.7 ([47](#_ENREF_47)) |
| Tait, Sheehy et. al. 2020 ([44](#_ENREF_44)) | Survey | Community pharmacist | Community Pharmacist | “One-fifth of community pharmacies carry formulations useful in managing terminal phase symptoms. The main factor associated with this was awareness of people with palliative needs using the pharmacy. Strategies that engage with pharmacists in anticipation of the terminal phase are critical, supporting people with palliative needs to remain at home to die, if desired.” Tait, Sheehy et. al. 2020 p.326 ([44](#_ENREF_44)) |
| Tait et. al. 2017 ([49](#_ENREF_49)) | Semi-structured focus group | Medical Specialist,  Pharmacist Specialist, Nurse Specialist, Medical Generalist, Pharmacist Generalist, Nurse Generalist. | Medical Specialist,  Pharmacist Specialist, Nurse Specialist, Medical Generalist, Pharmacist Generalist, Nurse Generalist. | “This study has identified six key themes, including: ‘Medication’ Supply’, ‘Education and Training’, ‘CaregiverBurden’, ‘Safety’, ‘Funding’ and ‘Clinical Governance’.” Tait et. al. 2017 p.377([49](#_ENREF_49)) |
| Thomas et. al. 2020 ([98](#_ENREF_98)) | Semi-structured interview. Transcripts were analysed using inductive thematic analysis. | General Practitioners, General Practitioner Trainees | General Practitioner | “This study found that GPs self-identify a responsibility to initiate end-of-life discussions, arising from their role and expertise, and these conversations’ benefits. This responsibility is modulated by clinical relevance, the doctor–patient relationship, and other healthcare providers’ involvement.” Thomas et. al. 2020 p.558 ([98](#_ENREF_98)) |
| To et. al. 2017 ([99](#_ENREF_99)) | Online survey | General Practitioners | General Practitioners | “Despite GPs’ beliefs of the benefits of case conferencing, the barriers remain significant. Enabling case conferencing will require support for organisation of case conferences and review of Medicare Benefits Schedule criteria for reimbursement.” To et. al. 2017 p.458 ([99](#_ENREF_99)) |
| Toye et. al 2015 ([100](#_ENREF_100)) | Action Research | Dementia patients | Nurse, Care Manager, Care Coordinator, Physician | “A palliative approach is widely recommended as a framework for care delivery to people who are drawing close to death with dementia and transfers across care settings can be anticipated.” Toye et. al 2015 p.554 ([100](#_ENREF_100)) |
| van de Mortel et. al. 2017 ([20](#_ENREF_20)) | Quasi-experimental design | Participants were referred by general practices and specialists to the palliative care service. Adults ≥18 years with a terminal illness were eligible to participate. | General Practice Registrar | “The results of this proof-of-concept pilot study demonstrated the feasibility of using a GP registrar to manage continuity of care for rural community palliative care patients.  This model has the potential to smooth out variations in GP availability and expertise through improved communication among the GPs, specialists, nurses and the patient and their family, which may provide superior continuity of care from multiple GPs.” van de Mortel et. al. 2017. p.142 ([20](#_ENREF_20)) |
